# Prediction of Altered 3′- UTR miRNA-Binding Sites from RNA-Seq Data: The Swine Leukocyte Antigen Complex (SLA) as a Model Region

**DOI:** 10.1371/journal.pone.0048607

**Published:** 2012-11-06

**Authors:** Marie-Laure Endale Ahanda, Eric R. Fritz, Jordi Estellé, Zhi-Liang Hu, Ole Madsen, Martien A. M. Groenen, Dario Beraldi, Ronan Kapetanovic, David A. Hume, Robert R. R. Rowland, Joan K. Lunney, Claire Rogel-Gaillard, James M. Reecy, Elisabetta Giuffra

**Affiliations:** 1 INRA, UMR 1313 de Génétique Animale et Biologie Intégrative, Domaine de Vilvert, Jouy-en-Josas, France; 2 CEA, DSV, IRCM, SREIT, Laboratoire de Radiobiologie et Etude du Génome, Domaine de Vilvert, Jouy-en-Josas, France; 3 AgroParisTech, Laboratoire de Génétique Animale et Biologie Intégrative, Domaine de Vilvert, Jouy-en-Josas, France; 4 Department of Animal Science and Center for Integrated Animal Genomics, Iowa State University, Ames, Iowa, United States of America; 5 Animal Breeding and Genomics Centre, Wageningen University, Wageningen, The Netherlands; 6 The Roslin Institute and Royal (Dick) School of Veterinary Studies, University of Edinburgh, Midlothian, United Kingdom; 7 Department of Diagnostic Medicine and Pathobiology, Kansas State University, Manhattan, Kansas, United States of America; 8 Animal Parasitic Diseases Laboratory, Beltsville Agricultural Research Center, Agricultural Research Service, United States Department of Agriculture, Beltsville, Maryland, United States of America; The Roslin Institute, University of Edinburgh, United Kingdom

## Abstract

The SLA (swine leukocyte antigen, MHC: SLA) genes are the most important determinants of immune, infectious disease and vaccine response in pigs; several genetic associations with immunity and swine production traits have been reported. However, most of the current knowledge on SLA is limited to gene coding regions. MicroRNAs (miRNAs) are small molecules that post-transcriptionally regulate the expression of a large number of protein-coding genes in metazoans, and are suggested to play important roles in fine-tuning immune mechanisms and disease responses. Polymorphisms in either miRNAs or their gene targets may have a significant impact on gene expression by abolishing, weakening or creating miRNA target sites, possibly leading to phenotypic variation. We explored the impact of variants in the 3′-UTR miRNA target sites of genes within the whole SLA region. The combined predictions by TargetScan, PACMIT and TargetSpy, based on different biological parameters, empowered the identification of miRNA target sites and the discovery of polymorphic miRNA target sites (poly-miRTSs). Predictions for three SLA genes characterized by a different range of sequence variation provided proof of principle for the analysis of poly-miRTSs from a total of 144 M RNA-Seq reads collected from different porcine tissues. Twenty-four novel SNPs were predicted to affect miRNA-binding sites in 19 genes of the SLA region. Seven of these genes (*SLA-1, SLA-6, SLA-DQA, SLA-DQB1, SLA-DOA, SLA-DOB* and *TAP1*) are linked to antigen processing and presentation functions, which is reminiscent of associations with disease traits reported for altered miRNA binding to MHC genes in humans. An inverse correlation in expression levels was demonstrated between miRNAs and co-expressed SLA targets by exploiting a published dataset (RNA-Seq and small RNA-Seq) of three porcine tissues. Our results support the resource value of RNA-Seq collections to identify SNPs that may lead to altered miRNA regulation patterns.

## Introduction

The swine major histocompatibility complex (MHC), also known as swine leukocyte antigen (SLA) complex, spans 2.4 Mb on swine chromosome 7 and is one of the regions of the swine genome with the highest gene density. More than 150 loci have been identified, with 129 of them annotated as protein-coding genes, including the classical and the non-classical MHC class I antigens and the MHC class II antigens [1]. An additional 124 ‘non-MHC genes’ are involved in natural immunity or have unknown functions. The Immuno Polymorphism Database-MHC (IPD-MHC) website (http://www.ebi.ac.uk/ipd/mhc/sla/) serves as a dedicated repository for maintaining a list of all SLA recognized genes and their allelic sequences [2].

As in MHC of other species, the swine MHC (SLA) region is highly polymorphic, with little recombination, producing large numbers of haplotypes segregating in populations [1], [3]. Several genetic associations with immunity and disease have been reported, as well as with production and reproduction traits [2]. However, most of the current knowledge on SLA is largely based on data from coding regions. Some sequence variants appear to regulate gene expression, rather than coding sequence [4]. A complex transcriptional pattern of non-classical SLA Ib genes, possibly due to post-transcriptional regulation, has been observed [5].

MicroRNAs (miRNAs) are small molecules (18–25 nt long) that post-transcriptionally regulate gene expression in metazoans. Most mammalian mRNAs (>60%) are predicted to be targeted by miRNAs, thus creating a complex layer of transcriptional repression that acts to diversify cellular phenotypes in a wide range of biological processes [6], [7]. Recent studies have revealed that miRNAs can often profoundly influence the response of fully developed tissues to physiologic and pathophysiologic stress, and that miRNAs play an important role in fine-tuning inflammatory mechanisms [8]. This functional niche indicates a central role for miRNA-regulated networks in disease states, which often represent an insufficient or aberrant response under conditions of stress or injury [9].

It is widely accepted that 3′ untranslated gene regions (3′-UTR) are the predominant location of miRNA target sites. In the canonical scenario, a perfect base-pairing complementarity between the critical “seed” region of the mature miRNA (nt 2–7) and the 3′-UTR of a target expressed gene leads to mRNA decay and/or translational inhibition; multiple binding sites for the same miRNA in 3′-UTRs can strongly enhance the degree of regulation [10], [11], [12]. Functional genomics and computational approaches are revealing additional complexity as well as alternative modes of miRNA and gene target recognition [13], [14]. An increasing number of bioinformatic strategies have been proposed to model miRNA-3′UTR interactions. Most methods require a perfect sequence complementarity between the miRNA seed region and the 3′-UTR. This has been supplemented by various filters taking into account additional criteria, including the phylogenetic conservation of miRNA recognition sites [6], [15], the thermodynamic stability of the putative miRNA:mRNA duplex [16], [17], and the structural features of the 3′-UTR region, in particular site accessibility or local context [18], [19], [10].

A DNA sequence polymorphism (DSP) in either the miRNA or its gene target sequence (poly-miRTS) may have a significant impact on gene expression by abolishing, weakening or creating miRNA-binding sites, and potentially lead to phenotypic consequences. For example, in the Texel sheep, the creation of miR-1 and miR-206 binding sites by a SNP in the 3′ UTR region of the *GDF8* shows perfect association with sheep hyper-muscularity [20], [21]. Genome wide catalogues of DSPs predicted to perturb miRNA-mediated gene regulation have been reported for miRNA target sequences of vertebrates (Patrocles database: www.patrocles.org; [22]) and for miRNA sequences [22], [23]. However, these predictions are based on perfect-seed matching of miRNA-binding sites and, furthermore, the pig species is not yet included in the Patrocles database [22].

We aimed to discover novel 3′-UTR variants in transcripts mapping to the whole SLA region, potentially leading to altered post-transcriptional regulation mediated by miRNAs, by taking into account different biological parameters to predict 3′-UTR miRNA target sites. We first explored the impact of SNPs on the 3′-UTR miRNA target sites of three SLA class I genes characterized by a different range of sequence variation. This provided proof of concept information for exploiting a collection of porcine RNA-Seq data from different individual animals and tissues. Finally, the analysis of a published ‘whole transcriptome’ deep sequencing dataset (RNA-Seq and small RNA-Seq) provided evidence of opposite expression levels between miRNAs and their co-expressed SLA targets.

## Results

### 3′-UTR Variants of SLA-1, SLA-3 and SLA-6

We first focused on three SLA genes known from previous studies to exhibit a different range of sequence variation. Coding variants within classical *SLA-1* (MHC class Ia antigen 1; 44 alleles), and *SLA-3* (MHC class Ia antigen 3; 26 alleles) are localized to exons 2 and 3, which form the class I protein peptide-binding groove. By contrast, the non-classical *SLA-6* (MHC class Ib antigen 6) is almost monomorphic (nine variants; [2]). By reference to the SLA sequence (haplotype Hp-1a.1) of the Vertebrate Genome Annotation (VEGA) database [24] and all available NCBI accessions, a set of non-redundant representative 3′-UTRs was compiled for the three genes. This set contained 32 unique 3′-UTR sequences from the 44 identified SLA-1 alleles, 17 3′-UTR sequences from the 26 SLA-3 alleles, and three 3′-UTR sequences from the nine SLA-6 alleles. The nucleotide variation of 3′-UTR sequences *of SLA-1* (∼39%) exceeded levels of variability at exon 2 and exon 3. The variation of 3′-UTR sequences of *SLA-3* and *SLA-6* was ∼12% and ∼2%, respectively (Table S1).

### miRNA Targets and Poly-miRTSs of SLA-1, SLA-3 and SLA-6

The combined use of three software programs (TargetScan, PACMIT, and TargetSpy) allowed us to take into account different biological parameters to predict 3′-UTR miRNA target sites, namely seed perfect matching and 3′-UTR local context (TargetScan), seed perfect matching and site accessibility (PACMIT), and 3′ compensatory sites (TargetSpy). The miRNA binding site conservation could not be used as a criterion for these genes, due to the absence of clear orthology in the human genome for SLA class Ia and Ib genes [1].

In order to categorize miRNA-binding sites that may be altered by SNPs, we qualified any sites absent in the 3′-UTR VEGA reference but present in one or more alleles as a ‘created site’. A ‘disrupted site’ was defined as a site present in VEGA but absent in one or more alleles. Short retrieved allele sequences may be either real variants or result from prematurely truncated sequencing, and would thus lead to overestimation of the number of disrupted sites and to underestimation of the number of created sites. Therefore, no miRNA binding site was considered as disrupted in the absence of sequence information.

**Figure 1:**
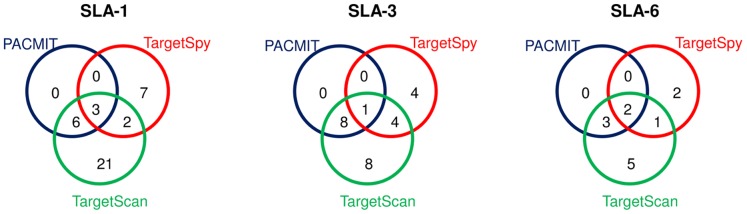
Venn diagrams of miRNA target sites at the 3′-UTRs of *SLA-1*, *SLA-3* and *SLA-6*. Predictions were performed by TargetScan, PACMIT and TargetSpy algorithms.

As expected, TargetScan predicted the highest number of miRNA target sites in the three genes (Table 1, Figure 1). A lower number of gene targets were predicted by PACMIT due to the additional constraint on site accessibility, all of which were common to TargetScan output, thus providing a first selection of TargetScan predictions based on site accessibility. TargetSpy was supposed to identify targets that were missed by TargetScan and/or PACMIT; indeed its output showed limited overlap with TargetScan and no overlap with PACMIT. Three, one, and two putative miRNA target sites were recognized by all three algorithms in *SLA-1, SLA-3, and SLA-6,* respectively. These predicted sites were jointly supported by a perfect seed match, 3-UTR local context, site accessibility, and 3′ compensatory sites criteria (Figure 1).

**Table 1:** miRNA-binding sites and poly-miRTSs (created and disrupted sites predicted by TargetScan, PACMIT and TargetSpy) in *SLA-1*, *SLA-3* and *SLA-6* porcine genes.

	TargetScan	PACMIT	TargetSpy
**VEGA sequence** a			
*SLA-1*	11	1	4
*SLA-3*	10	1	3
*SLA-6*	10	5	3
**Disrupted sites**			
*SLA-1*	10	1	4
*SLA-3*	6	1	3
*SLA-6*	1	0	3
**Created sites**			
*SLA-1*	21	8	8
*SLA-3*	11	8	6
*SLA-6*	1	0	2

a The VEGA sequence is the reference haplotype Hp-1a.1.

**Figure 2:**
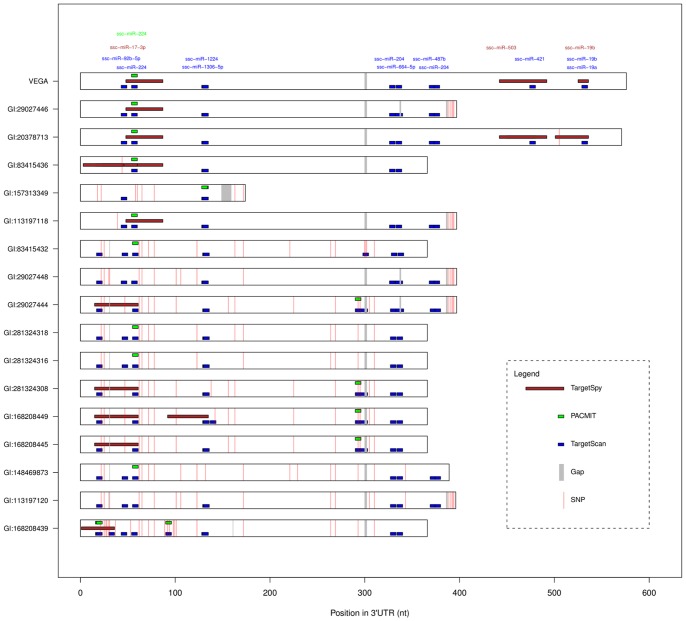
Influence of sequence variants on predicted miRNA target sites at the 3′-UTRs of SLA-3. Known alleles (17 sequences) were retrieved from NCBI database. TargetScan (blue), PACMIT (green), TargetSpy (brown). The miRNAs targeting the VEGA reference are indicated in text. The complete list of miRNAs targeting the altered target sites in SLA-3 alleles is reported in Table S2.

To distinguish “created” versus “disrupted” miRNA-targeting sites, our strategy needed to define a reference. Although the VEGA sequence was arbitrarily chosen, it turned out that the miRNA-binding sites predicted on VEGA were the most frequent in the alleles of the three genes. Based on this reference, all software programs predicted an excess of creation vs. disruption of miRNA target sites in the three genes (Table 1), and in several cases the same SNPs created and disrupted a targeting site for a different miRNA. These features are graphically illustrated in the case of *SLA-3* (Figure 2). A prevalence of created vs. disrupted sites has been reported in humans, leading to the hypothesis that a stronger purifying selection acts against SNPs destroying target sites than against SNPs creating target sites [25].

All the miRNA-binding sites predicted on the VEGA reference sequence were present in at least one of the described alleles of each gene (Table S2) and poly-miRTSs were predicted both in variable and highly conserved portions of the 3′-UTR regions of the three genes. Each poly-miRTS was either found in several alleles (e.g. miR-224 site in *SLA-3*), or in a single allele (e.g. miR-216 site in *SLA-3*: gb_EU432082.1 allele) (e.g. *SLA-3*: Figure 2). The number of poly-miRTSs reflected the different levels of 3′-UTR nucleotide variation (Table S1), with few poly-miRTSs found in *SLA-6*.

### Collection and Mapping of Porcine RNA-Seq Reads

A total of 144 M porcine RNA-Seq reads were obtained from alveolar macrophages (from a 10 week old Large White x Landrace cross pig), placenta (from a Duroc clone TJ Tabasco x Duroc cross pig), a pool of eleven tissues (from a three year old clone of TJ Tabasco pig), testis (from an European wild boar) and whole blood (from two commercial crossbred pigs). Reads were mapped against a custom swine genome (Table 2).

**Table 2:** Mapping statistics (number of reads) of 144 M RNA-Seq reads obtained from different pig tissues.

	Alveolar macrophages	Pool of fetal tissues	Placenta	Testis	Whole blood
**Reads located in the SLA region**	1177825	97651	101556	126306	268162
**Number of 3′-UTRs covered** a	69	84	59	73	102
**Total mapped**	31487788	14617543	10063066	8306799	41488609
**Proper pairs**	22786379	11397820	8169118	6868205	-
**Total reads**	36168380	38116682	11620273	9608149	48973230

a Number of 3′-UTRs mapping to the SLA region with more than 50% of the sequence covered by at least one read.

As expected, the proportion of reads that mapped to the SLA region varied across the different tissues. Alveolar macrophages had the highest proportion of reads (3.7%) that mapped to the SLA complex, reflecting their known high expression of both class I and class II MHC genes and several inducible non-classical MHC products such as TNF-alpha (DAH, RK, unpublished).

As we were interested in identifying the largest set of polymorphisms in the 3′-UTRs of the SLA region, we looked at the fractions of the 3′-UTR sequences covered by at least one read. Among the 151 genes localized in the region, 118 had 3′-UTR that were annotated and, of those, 111 genes had a non-zero coverage for more than 50% of their 3′-UTR sequence (Table 2).

### Analysis of DNA Sequence Polymorphisms (DSPs) from RNA-Seq Data

A total of 44 SNPs were identified in the 3′-UTRs of 25 genes in the SLA region (Table S3), of which 42 were not previously submitted to dbSNP. Only ten of the 44 SNPs were common to multiple datasets. This low degree of overlap is probably due to the stringent SNP calling criteria applied (see Material and Methods) and to the different depths of RNA-Seq coverage in these regions, and possibly to the effect of different breed of origin of individual animals (Table S3).

The complexity of the SLA region due to repeats, pseudogenes and paralogous gene families makes accurate mapping of short reads potentially difficult. The concept of sequence mappability has been introduced to discern, given the length of the sequenced reads and the number of mismatches allowed during the mapping step, the regions producing reads which map back unambiguously to themselves [26]. We computed the mappability of each of the 44 identified SNPs based upon the technical specifications of the sequencing experiment from which they originate. Only six out of the 44 SNPs had a mappability greater than 1, meaning that reads covering them could map more than once on the genome (Table S3). Therefore, only the 38 remaining SNPs were considered in further analysis.

### Effect of Sequence Polymorphism on Predicted miRNA Target Sites

Poly-miRTS in RNA-Seq data were evaluated using the same combined approach of target site prediction (TargetScan, PACMIT and TargetSpy software). A total of 46 3′-UTR SNPs were taken into account, which included the 38 SNPs identified from RNA-Seq datasets, plus eight known SNPs previously reported in the dbSNP database.

More than half of these SNPs (24 out of 46) were annotated as potentially altering miRNA binding in a total of 19 genes of the SLA region (Table S4). As previously found for SLA-1, SLA-3 and SLA-6 known alleles (Table 1), all three software programs predicted an excess of created miRNA target sites, and often the same SNPs created a new site while disrupting a targeting site for a different miRNA. For example, both TargetScan and PACMIT predicted that the DSP in the *ABCF1* 3′-UTR created a new ssc-miR-34a/c target site while disrupting the ssc-miR-885-3p predicted target site (Table S4).

While PACMIT confirmed most (17 out of 23) of the created sites predicted by TargetScan, pronounced differences were found for disrupted sites (4 common sites out of 13 sites predicted by both software applications). This can be explained by the additional accessibility criterion of PACMIT as sites considered as created or disrupted by PACMIT may only reveal changes in secondary structures which are not predicted by TargetScan (e.g. the created ssc-miR-339-5p and ssc-miR-4334-3p sites in *CREBL1* 3′-UTR, and the disrupted ssc-miR-148a, ssc-miR-148b and ssc-miR-152 sites in *HSPA1A)*.

As expected, the output of TargetSpy (whose main criterion is the alteration of 3′ compensatory sites) was considerably different from TargetScan and PACMIT. For example, only TargetSpy predicted the creation of a ssc-miR-376a site in *PPP1R10* and the disruption of a site for ssc-miR-181c in *SLA-DRA* (Table S4). Two created sites, ssc-miR-19a and ssc-miR-19b targeting *HSPA1A* (encoding the heat shock 70 kDa protein 1A), found by TargetScan and PACMIT overlapped with TargetSpy predictions.

### Conservation of Predicted miRNA Target Sites in Humans

A consideration of inter-specific conservation of miRNA target sites can significantly improve target prediction, the rationale being that highly conserved miRNA-binding sites are more likely functional. Among the three chosen algorithms, only TargetScan is implemented to exploit this filter.

Out of the 13 genes in which polymorphic miRNA target sites were predicted by TargetScan, 11 had a human ortholog. At three of these genes, we identified miRNA-binding sites conserved between pigs and humans (Table S4). In particular, the miR-139-5p target site in *SLA-DQB1* was conserved in *HLA-DQB1*, and we found a variant that disrupted this site in the pig. Three target sites were predicted for miR-1296 in the 3′-UTR of both human and pig *DDR1,* with one out of the three sites altered in one pig variant (i.e. one disrupted poly-miRTS). Finally, the miR-423-5p target site in the 3′-UTR of human *RNF5* was conserved in one porcine variant (i.e. one ‘created’ poly-miRTS) (Table S4).

### Co-expression Patterns of miRNAs and Poly-miRTSs from Porcine RNA-Seq and Small RNA-Seq Data

The biological relevance of predicted poly-miRTS (Table S4) would require validation on a case by case basis, as it is directly dependent on the co-expression patterns of the miRNA and its predicted target in the same tissue and at a given physiological state [27]. Consequently, we reasoned that preliminary information on the relationship between poly-miRTSs and their cognate miRNAs expression levels could be inferred by the joint analysis of available RNA-Seq and small RNA-Seq data generated from the same cells or tissue. Poly-miRTSs may either determine acquisition or loss of a miRNA target for a given gene. Thus, expressed gene variants harboring a poly-miRTS for any co-expressed miRNAs should be represented by a lower (if the target site is created) or higher (if the site is disrupted) number of reads than the reference allele.

We analyzed the RNA-Seq and small RNA-Seq expression profiles of three porcine tissues (abdominal fat, liver and longissimus dorsi muscle obtained from two individual pigs) of a recently published study [28]. The raw reads from the six RNA-Seq libraries and the six small RNA-Seq libraries were mapped against the custom swine genome, and transcripts and miRNAs expression levels were quantified. The 19 previously identified genes in the SLA region harboring poly-miRTSs (Table S4) were expressed in at least one of the tissues, and more than 44 potential mRNAs and cognate miRNAs pairs showed evidence of co-expression (Figure S1).

Although this study evaluated only two individuals, we expected a high degree of polymorphism as these animals had been obtained by an F2 inter-cross of divergent breeds [28]. Indeed, we could identify 16 out of the 24 poly-miRTSs previously identified in the 19 expressed genes (Table S4).

**Figure 3:**
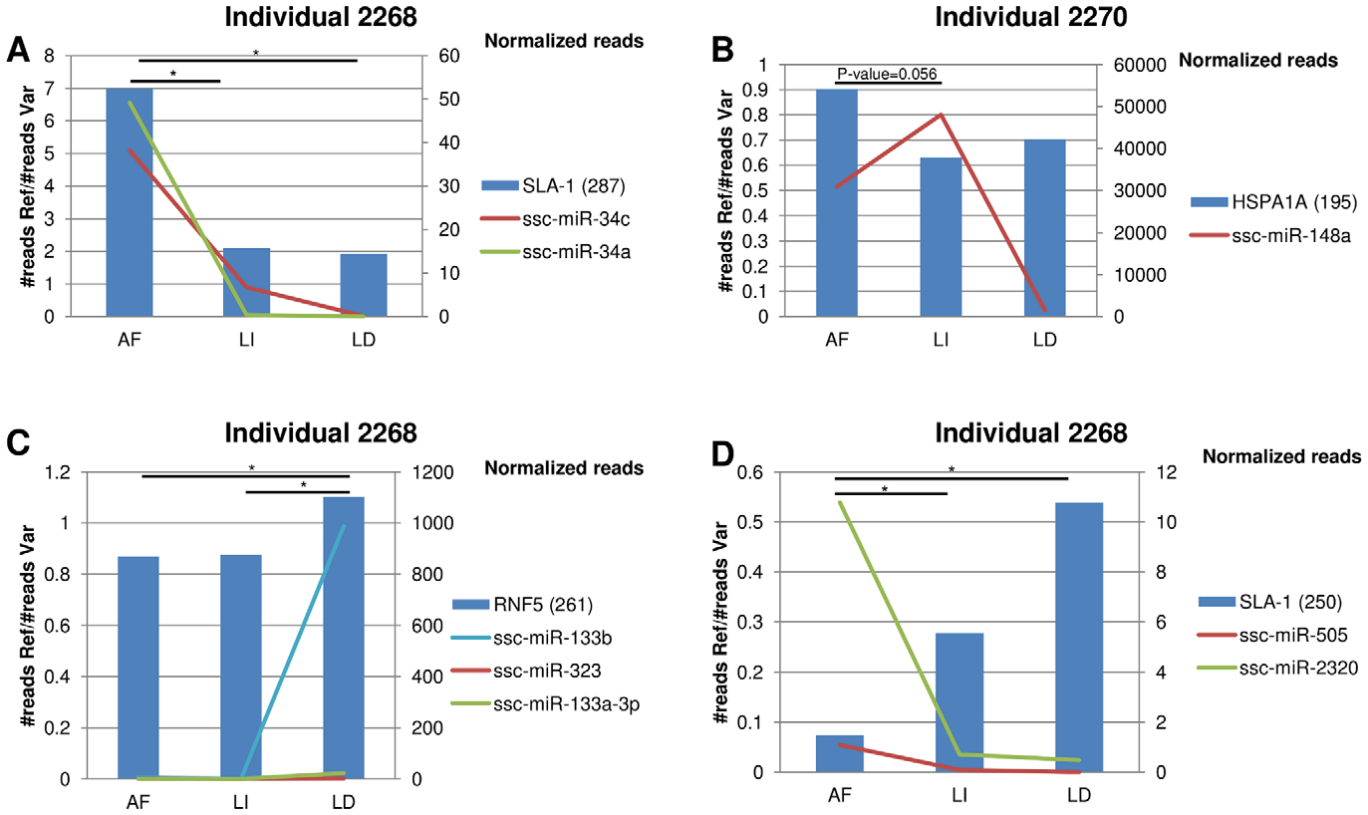
Co-expression patterns of four poly-miRTSs and their cognate miRNAs. RNA-Seq and Small RNA-Seq reads were obtained from abdominal fat (AF), liver (LI) and longissimus dorsi muscle (LD) of two individual pigs (pig 2268 and pig 2270). Left y-axis: ratios of the number of reads of reference allele vs. variant allele at poly-miRTSs positions; right y-axis: expression levels of targeting miRNAs. Numbers in parenthesis indicate the position of the poly-miRTSs in the 3′-UTR. The asterisks indicate significant differences between tissues (p-values <0.05).

We focused on the subset of genes expressing both the reference allele and a variant carrying a poly-miRTS, and for which the analysis of reads indicated different levels of allele specific expression between at least two tissues. Four poly-miRTSs in three genes (*SLA-1, HSPA1A* and *RNF5)* predicted to be targeted by a total of 13 co-expressed miRNAs fulfilled these criteria (Figure 3, Table S5).

At these three genes, we found evidence of inverse expression of the alleles bearing the poly-miRTS and seven cognate miRNAs. This pattern included the two created target sites for ssc-miR-34a and ssc-miR-34c, both predicted by TargetScan and PACMIT in *SLA-1* (Figure 3 A); the disrupted target site for ssc-miR-148a in *HSPA1A* predicted by PACMIT and TargetSpy (Figure 3 B); the ssc-miR-133b (TargetScan and PACMIT), ssc-miR-133a-3p (TargetScan) and ssc-miR-323 (TargetSpy) created target sites in *RNF5* (Figure 3 C); and the disrupted site for ssc-miR-2320 predicted by TargetSpy in *SLA-1* (Figure 3D).

### Allele-specific Expression at Poly-miRTS Positions

**Figure 4:**
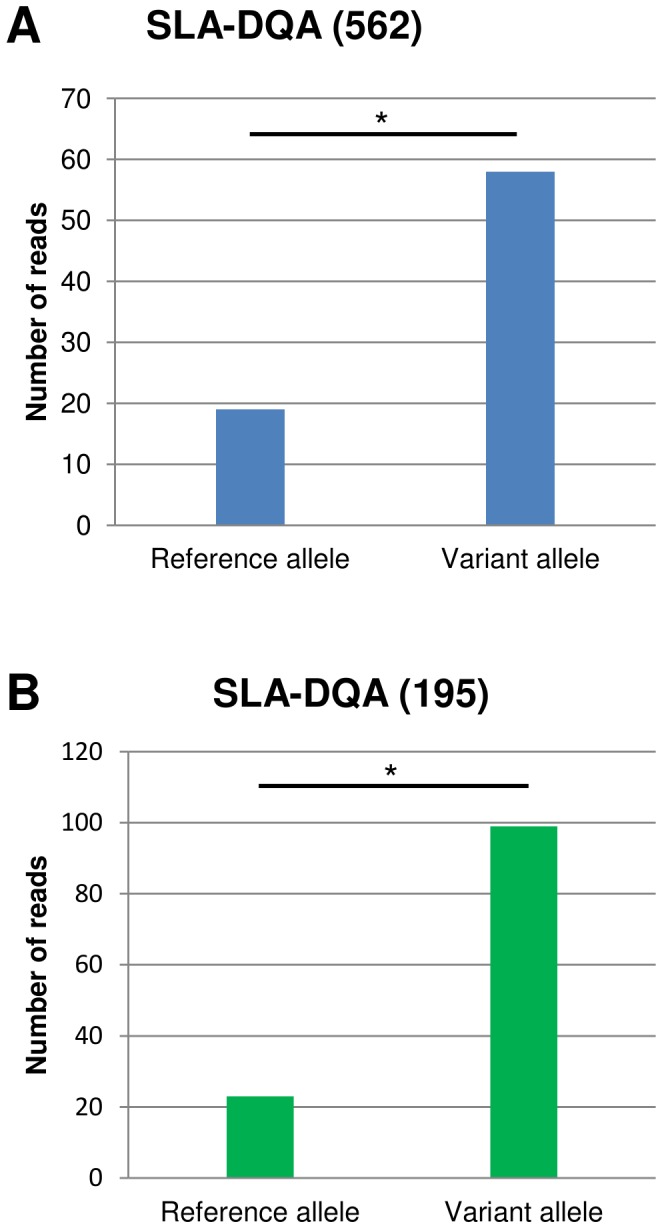
Allele-specific expression at two poly-miRTSs in *SLA-DQA*. RNA-Seq reads were obtained from alveolar macrophages (A) and whole blood (B) libraries. The asterisk indicates a significant difference between reads bearing the reference and the variant alleles assessed by a binomial test, p-value <0.001.

We searched for evidence of allele-specific expression at poly-miRTSs in the previously analyzed RNA-Seq libraries. Interestingly, two poly-miRTSs localized in regions of the 3′-UTR of *SLA-DQA,* with a mappability of one, showed allele specific expression. One of them was observed in the alveolar macrophage library, while the other was found in one of the whole blood samples. In both cases, the variant allele was significantly represented by more reads than the reference alleles (Figure 4). The predicted targeting miRNAs of *SLA-DQA* are known to be co-expressed with HLA-DQA1 in human whole blood [29], [30].

## Discussion

### Extensive Predictions of Poly-miRTSs

TargetScan, PACMIT and TargetSpy allowed us to predict potential miRNA 3′-UTR target sites, and ultimately to discover potential poly-miRTSs, by multiple criteria. In addition to the canonical seed pairing mechanism, site secondary structure can influence the strength of miRNA regulation [31] and SNPs located outside the seed region may influence target expression [32], [33]. Moreover, although 3′ compensatory sites are considered to be rare, there is functional evidence of their importance. Matching of the miRNA’s nt 13–17 can compensate for a single-nucleotide bulge or mismatch in the seed region, as illustrated by the experimentally validated let-7 sites in *LIN41* [34] and the miR-196 site in *HOXB8* [35]. Furthermore, 3′ compensatory sites could be a way to favor regulation by a specific miRNA which belongs to a family with members sharing the same seed [36].

The combination of multiple algorithms for miRNA target prediction provides a better specificity (i.e. an higher probability to detect true negatives) but a lower sensitivity in predicting real target sites [37]. In the context of an exploratory analysis of a large genomic region, it would seem more relevant to minimize the number of false predictions, even at the cost of missing some of the true target sites. A high level of precision (∼50%) may be obtained by taking into account the criterion of inter-specific conservation in addition to perfect seed pairing (as implemented by TargetScan), but this approach alone would miss the most numerous non-conserved target sites (sensitivity: ∼12%) [37]. PACMIT has a slightly lower performance in terms of precision (sensitivity: 20%, and precision: 40%) but it permits consideration of accessibility and perfect seed matching independently of conservation [19]. Finally, although identifying 3′ compensatory sites without filtering by perfect seed pairing is difficult, as evident from the low performance of most tools that allow identification of these sites, TargetSpy has been described as the best available approach for identifying such sites [38].

The predictions on *SLA-1*, *SLA-3* and *SLA-6* (Table 1, Figure 1, Figure 2), characterized by a different range of sequence variation, provided proof of concept information for this combined approach, encouraging further analysis of poly-miRTSs from RNA-Seq data. Furthermore, this allowed investigating co-occurring SNPs that could impact several miRNA target sites. As illustrated in Figure 2, one of the poly-miRTSs identified by TargetSpy (a created site for miR-224 at position 50 of *SLA-3* 3′-UTR) was always associated with a miR-27a/b created site predicted by TargetScan and PACMIT at position 298. These three miRNAs are expressed in human placenta [39], indicating that they may act cooperatively in that tissue.

### Predictions of Poly-miRTS in RNA-Seq Data

The analysis of 144 M RNA-Seq reads permitted the systematic exploration of the 3′-UTR variability of the whole SLA complex, which is so far one of the best characterized and annotated regions of the swine genome [40]. TargetScan, PACMIT and TargetSpy predicted a total of 24 novel poly-miRTSs in 19 genes mapping to the region, including seven genes (*SLA-1, SLA-6, SLA-DQA, SLA-DQB1, SLA-DOA, SLA-DOB* and *TAP1*) linked to antigen processing and presentation functions (Table S4). In humans, altered miRNA binding to MHC class I classical and non-classical genes has been associated with disease control or susceptibility. Allele-specific targeting of *HLA-G*, a non-classical HLA class I locus, by miR-148a and miR-148b, is associated with risk of asthma [41]. Kulkarni et al. [42] reported a SNP in the 3′-UTR of the classical HLA class I molecule, *HLA-C*, associated with HIV control, directing miRNA-mediated regulation of HLA-C allotypes. Both studies emphasize the potential role of regulatory regions in order to obtain a comprehensive view of MHC variability [43]. The same argument is obviously valid in livestock species. In this context, the combined predictive approach is of particular relevance for the SLA gene complex. Only a fraction of the porcine SLA genes have known orthologs in humans and other model genomes, thus limiting the possibility to use the criterion of inter-specific conservation of miRNA target sites.

### Co-expression Patterns of miRNAs and Poly-miRTSs in Porcine RNA-Seq and Small RNA-Seq Data

The reanalysis of ‘whole transcriptome’ public sequencing data from three porcine tissues (RNA-Seq and small RNA-Seq) [28] allowed us to obtain a global picture of the co-expression of 16 predicted poly-miRTS of the SLA region and at least one of their respective cognate miRNAs (Figure S1, Figure 3, Table S5). Co-expression is one of the fundamental criteria for functionality of predicted miRNA-mRNA interactions which concerns both evolutionary conserved and non conserved miRNA-mRNA interactions. In humans, it has been estimated that 30%–50% of non conserved miRNA target sites are functional when the mRNA and miRNA are endogenously co-expressed [44].

For two of the three miRNA-target interactions conserved in humans in which we have identified a poly-miRTSs in pig (see Results *Conservation of predicted miRNA target sites in humans)*, we could find evidence of co-expression in the three porcine tissues. In particular, ssc-miR-139-5p and its putative target *SLA-DQB1* were co-expressed in the three tissues, and ssc-miR-423-5p and its putative target *RNF5* were co-expressed in Abdominal Fat and Longissimus Dorsi muscle. No functional validation has been reported for these interactions in other species. However, available information in humans and mice point to evidence of co-expression in spleen and in the lymphoid lineage [30], [45]. Although our observations represent only a subset of genes, tissues and physiological conditions, and cannot take into account other cis- and trans- factors, the analysis of the whole transcriptome data provided significant examples of inverse expression levels between the expression levels of targeted alleles and their cognate miRNAs. Such matches of expression profiles were found for four (three created, one disrupted) of the nine predictions supported by multiple algorithms, while only one out of the eight sites predicted by a single algorithm was consistent (Figure 3). Although miRNA-mediated regulation does not always lead to a change in mRNA abundance [46], these results suggest that predictions supported by multiple algorithms are more reliable for genome wide predictions of poly-miRTSs, especially when the inter-specific conservation criterion is missing.

### Conclusions

The increasing amount of RNA-Seq data generated in pigs and other livestock can be used as a resource to identify SNPs that potentially impact miRNA regulation, particularly for genome wide association studies of disease and ‘robustness’ traits. There is high genetic diversity segregating in livestock breeds in spite of artificial selection and, as expected, several novel miRNAs have been described in livestock (e.g. [47]). Although most miRNA target sites are usually considered under strong purifying selection in humans due to their impact on disease traits, a study in cichlid species has suggested that diversification of miRNA targets may be an important evolutionary mechanism of phenotypic diversification and speciation (reviewed by [48]). The forthcoming availability of well-annotated genomes and data from ‘1000 genomes’ and ENCODE projects in animals will be a fundamental step for the further implementation of combined poly-miRTS predictions, as well as of co-expression analyses of poly-miRTSs and miRNAs on the genome wide scale.

## Materials and Methods

### SLA Sequences

We used annotation and sequences from the Vertebrate Genome Annotation database (VEGA) (Release 44) for the SLA region as a reference. Reference 3′-untranslated region (3′-UTR) locations were considered as annotated in VEGA database. VEGA database [24] provides a high quality manual annotation of specific regions or entire genomes of vertebrate species. Sequences of additional alleles of the SLA class I genes (*SLA-1, SLA-3* and SLA-6; Table S1) were retrieved from NCBI RefSeq database [49].

### miRNAs Target Prediction

TargetScan algorithm was used to predict messenger RNAs targeted by microRNAs (miRNAs) [10] The specificity of this approach is based on the calculation of a context score for the 3′-UTRs. This score takes into account five additional features of 3′-UTRs outside of the seed complementarity that influence miRNAs binding site efficiency. TargetScan can use information of site inter-specific conservation to strengthen predictions. However, no clear human orthologs have been identified for SLA class I genes [2] and, in consequence, this criterion was restricted to genes for which we could find human orthologs.

PACMIT proposes to complement matching of the miRNAs seed region and site accessibility with a ranking strategy relying on over-representation [19]. The rationale for considering site accessibility is that the secondary structure of 3′-UTRs may facilitate or prevent miRNA binding. The criterion to consider a site ‘accessible’ is that at least four nucleotides of the site complementary to the seed (nt 2–8 of the miRNA) are in an open loop structure. The predictions are then ranked according to over-representation assuming that functional targets should contain complementary sites that are over-represented among the accessible ones. TargetSpy implements a supervised machine learning algorithm for identification of miRNAs targets [38]. This approach allows the prediction of miRNA-binding sites without seed perfect complementarity. It relies on a set of discriminative features to be used for machine learning. Like PACMIT, TargetSpy is able to identify species-specific target sites independent of site conservation.

### Poly-miRTS Prediction

A miRNA binding site was considered as a polymorphic miRNA target site (Poly-miRTS) when there was a different miRNA prediction for the reference and the variant sequences. miRNA target sites predicted by one or several algorithms in the reference sequence, but not present in the variant sequence were considered as disrupted sites. miRNA target sites predicted by one or several algorithms in the variant sequence, but not in the reference sequence were considered as created sites.

We also considered the score attributed by each algorithm to identify perturbed, but not disrupted, miRNA-mRNA interactions. For TargetScan algorithm, we used the context score given for each miRNA target sites, which is based on several UTR features, local AU content, miRNA binding site position and site type, whereas for PACMIT and TargetSpy, we used the score which was associated with each prediction. Thus, a SNP is perturbing UTR context when there is a difference of >2 units between the reference and the variant UTR scores.

### RNA-seq Datasets

RNA-Seq reads (Illumina) were collected from different experiments (Table 2).

#### Alveolar macrophages

Cells were collected from a 10 week old Large White x Landrace cross pig. The pig was sedated with a mixture of azaperone (1 mg/kg) and ketamine (6 mg/kg) and left undisturbed for a minimum of 15 min before being killed by captive bolt. Trachea was clamped, lungs were harvested and washed twice with 500 mL of PBS in sterile environment. The resultant bronchoalveolar washes were filtered (100 microns) and spun at 400 g for 10 min. These alveolar macrophage cells were resuspended in a freezing medium (90% heat-inactivated FCS, 10% DMSO) and frozen overnight in a ‘Mr. Frosty’ isopropanol box at −80°C (Nalgene) allowing a controlled decrease of temperature. The next day, cells were transferred to a −150°C freezer for long-term storage. The alveolar macrophages were quickly thawed and cultured as in [50] with minor modifications. Briefly, cells were washed with PBS to remove DMSO and cultured in complete medium-RPMI 1640, 10% heat-inactivated FCS, penicillin/streptomycin, and GlutaMAX-I supplement (Life Technologies), without addition of rhCSF-1. The next morning non adherent cells were removed. Adherent macrophages were stimulated with LPS (100 ng/ml) for 7 h. Total RNA was extracted with the RNeasy mini kit (Qiagen) following manufacturer’s instructions and stored at −80°C until used. The Illumina mRNA-seq Sample Preparation Kit was used for sample preparation (∼5 ug of total RNA) following manufacturer’s instructions (Illumina, Part # 1004898 Rev. D). RNA quality and yield after sample preparation were affirmed by Agilent Bioanalyzer analyses. Library size was ∼200 bp. Sequencing of the library was performed on an Illumina Genome Analyzer IIx and generated 35 bp paired-end reads.

All animal care and experimentation procedures were conducted in accordance with the guidelines of Roslin Institute and the University of Edinburgh and under Home Office Project License PPL 60/4259.

#### Placenta

The placenta was collected at day 113 of fetal development/pregnancy from a Duroc (clone TJ Tabasco)×Duroc cross pig. Total RNA was extracted with the RNeasy mini kit (Qiagen) following manufacturer’s instructions and stored at −80°C until used. The Illumina mRNA-seq Sample Preparation Kit was used for sample preparation (∼5 ug of total RNA) following manufacturer’s instructions (Illumina, Part # 1004898 Rev. D). Quality and yield after sample preparation was measured with a DNA 1000 Lab-on-Chip (Agilent Technologies, Inc.). Library size was ∼200 bp. Sequencing was performed on the Illumina Genome Analyzer IIx and generated 51 bp paired-end reads.

#### Pool of eleven tissues

Ten tissues (colon, kidney, hypothalamus, spleen, small intestine, lymph node, liver, lung, frontal lobe, cerebellum) were obtained from a three year old clone of TJ Tabasco (the mother in the cross above), plus the placenta sample (thus in total 11 tissues). Total RNA was extracted from each sample separately with the RNeasy mini kit (Qiagen) following manufacturer’s instructions, pooled in equally molar ratios and stored at −80°C until used. The pooled RNA sample was used for ‘Full-length cDNA library cloning’ and prepared for Illumina GA IIx sequencing at DNAFORM/RICKEN Institute, Japan (http://www.dnaform.jp/products/cdna_e.html). Sequencing generated 51 bp paired-end reads. The sample collection protocol for the preparation of the ‘placenta and ‘pool of eleven tissues’ libraries was approved by the Institutional Animal Care and Use Committee of the University of Illinois at Champaign-Urbana, USA.

#### Testis

This sample was obtained from a one year old European wild boar (sampled at the Veluwe, The Netherlands). Total RNA was extracted with the RNeasy mini kit (Qiagen) following manufacturer’s instructions and stored at −80°C until used. The Illumina mRNA-seq Sample Prep Kit was used for sample preparation (∼5 ug of total RNA) following manufacturer’s instructions (Illumina, Part # 1004898 Rev. D). Quality and yield after sample preparation was measured with a DNA 1000 Lab-on-Chip (Agilent Technologies, Inc.). Library size was ∼200 bp. Sequencing was performed on the Illumina Genome Analyzer IIx and generated 51 bp paired-end reads. The sample collection protocol for the preparation of the testis library was approved by the Institutional Animal Care and Use Committee of the University of Wageningen, The Netherlands.

#### Whole blood

Blood samples were collected using Tempus™ Blood RNA Tubes (Applied Biosystems) from two commercial crossbred pigs at Kansas State University as part of the PRRS Host Genetics Consortium (http://www.animalgenome.org/lunney/index.php). Samples were stored at −20°C until ready for RNA extraction. Thawed samples were processed following the Spin RNA isolation protocol (Applied Biosystems, Part # 4329232 rev. D). RNA quality and yield were affirmed by Agilent Bioanalyzer analyses. The TruSeq RNA Sample Prep Kit was used for sample preparation (∼5 ug of total RNA) according to the TruSeq Sample preparation guide (Illumina, Part # 15008136 Rev. A). Sequencing was performed on the Illumina Genome Analyzer IIx and generated 70 bp single-end reads.

The Kansas State University Institutional Animal Care and Use Committee approved all experimental protocols for this study.

All the RNA-Seq datasets have been submitted to the NCBI Sequence Read Archive (SRA) under accession numbers: SRA057401 (alveolar macrophages), SRA057367 (placenta and pool of tissues) and SRA057414 (whole blood).

### Read Mapping

Before mapping, reads were trimmed on quality, adaptors and poly(A) tail using BRAT [51] and custom Perl scripts.

Reads were mapped with Tophat v.1.3.1 [52] against a pig custom reference genome with the SLA region sequence and annotation obtained from the VEGA database (release 44); the remaining sequences and annotation were from Ensembl release 61 (Sscrofa9.2 sequence). The following Tophat settings were used: maximum alignments per read were set to 1, expected mean inner distance between mate pairs of 100 and other parameters set to default.

To get an overview of the coverage of the 3′-UTRs in the SLA region, each of the 118 3′-UTRs annotated in VEGA database was qualified as ‘covered’ when more than 50% of its sequences was covered by at least one RNA-Seq read.

### SNP Identification

Before SNP identification, Picard tools (http://picard.sourceforge.net) were used in order to remove duplicate reads that may lead to overestimation of read depth and SNP misannotation.

SNPs were identified from individual samples using Samtools [53] mpileup function on Tophat mapped reads. The minimum SNP quality was set to 50 and the minimum read depth to 15. Only SNPs in annotated 3′-UTRs of genes located in the SLA locus were retained for further analyses.

### Mappability

Concept and implementation procedures of the mappability method are fully described in [26]. Briefly, the rationale is to identify the regions of the genome which are truly ‘mappable’, i.e. producing reads which map back uniquely to their regions of origin. Computing mappability for a given genome mostly depends on the length of the sequence reads produced by the experiment, and on the number of mismatches allowed during the mapping step.

We estimated the mappability of the custom genome sequence using the ‘gem-mappability tool’, according to each different technical specification of the RNA-Seq experiments (read length parameter of 90 bp, 70 bp, 50 bp and 35 bp). The number of mismatches was set to 2 for each estimation.

To assess mappability of poly-miRTS locations, we averaged the mappability of each SNP position within a 2× read length region surrounding the poly-miRTS. Only the poly-miRTSs with an average mappability equal to 1, i.e. those on which reads tend to map back uniquely, were retained.

### SNPs from Public Databases

We retrieved annotated SNPs in this region from dbSNP database (release136) and the porcine Illumina 60 K SNP chip [54]. Eight SNPs belonged to the 3′-UTR region of 7 genes of the SLA locus (genes and SNPs accession IDs are summarized in table S6).

### miRNA and Predicted Target Co-expression

Chen et al. [28] have described the whole transcriptome (mRNA and miRNA expression) from two full-sib Duroc×Erhualian F2 individuals with extreme growth and fat phenotypes. To investigate allelic differential expression, we retrieved the raw data of this study from NCBI Gene Expression Omnibus under accession no. GSE26572 (small RNAs and mRNAs). About 118 M reads corresponding to the six mRNA libraries were processed (trimming and mapping) as described above. Additionally, transcripts were assembled and quantified by Cufflinks v2.0.0 [55]. Cufflinks provides expression levels using an estimation of fragments per kilobase of exon per million reads mapped (FPKM). More than 67 M reads sequenced from the six small RNA libraries were trimmed for low-quality ends (cutoff 20) and adapters using cutadapt [56] and mapped with Bowtie [57] against the pig custom genome reference. A maximum of 2 mismatches were allowed. In order to avoid discarding of identical mature sequences from distinct precursors, reads mapping at a maximum of 5 different positions on the genome were retained.

We considered as co-expressed a miRNA represented by more than 10 reads and a mRNA with more than 1 FPKM.

To examine miRNA and cognate mRNA expression levels across tissues, we plotted the allelic proportions at the poly-miRTS sites and the expression levels of the cognate miRNAs. We focused on poly-miRTSs showing different allelic proportions (ratios of the number of reads of reference allele vs. variant allele at poly-miRTS positions) between at least two tissues. To assess the statistical significance of these differences, we assigned a p-value (Fischer’s exact test) to the pairwise comparisons of allelic proportions between tissues at (inferred) heterozygous poly-miRTS sites.

### Allelic Specific Expression

For all poly-miRTSs, we performed a two-sided binomial exact test of the null hypothesis that the variant and reference read counts are equal (i.e. binomial success probability = 0∶5). We used the “binom.test” function in R [58] to carry out this test for each gene and chose a P-value threshold of 0.001.

## Supporting Information

Figure S1 Venn diagrams of miRNA target sites predicted in pig 2268 and pig 2270. Predictions were performed on genes expressed in abdominal fat (AF), liver (LI) and longissimus dorsi muscle (LD) by TargetScan, PACMIT and TargetSpy algorithms.
(TIFF)

Table S1 Nucleotide variability of *SLA-1*, *SLA-3* and *SLA-6*, and accession numbers of the retrieved SLA-1, SLA-3 and SLA-6 alleles.
(XLSX)

Table S2 miRNA target prediction in SLA-1, SLA-3 and SLA-6 alleles by TargetScan, PACMIT and TargetSpy. Underlined microRNAs were predicted by both TargetScan and PACMIT.
(XLSX)

Table S3 SNPs identified in the SLA gene complex using a RNA-Seq data collection from different tissues. The “Y” indicates a SNP passing the identification filters.
(XLSX)

Table S4 List of created and disrupted miRNA target sites in the SLA region predicted from RNA-Seq data. Target sites conserved in humans are marked by #. Target sites with perturbed but not disrupted structure are designated by (s).
(XLSX)

Table S5 Number of reads corresponding to the reference and the variant alleles at poly-miRTSs positions in abdominal fat, liver and longissimus dorsi muscle.
(XLSX)

Table S6 Accession numbers of the eight known SNPs within 3′-UTRs of genes of the SLA region retrieved from dbSNP database.
(XLSX)
