# Supplementary figures and images for: Prediction of Altered 3′- UTR miRNA-Binding Sites from RNA-Seq Data: The Swine Leukocyte Antigen Complex (SLA) as a Model Region
Source: PLoS One. 2012 Nov 6;7(11):e48607. doi: 10.1371/journal.pone.0048607 (PMC3490867; doi:10.1371/journal.pone.0048607)

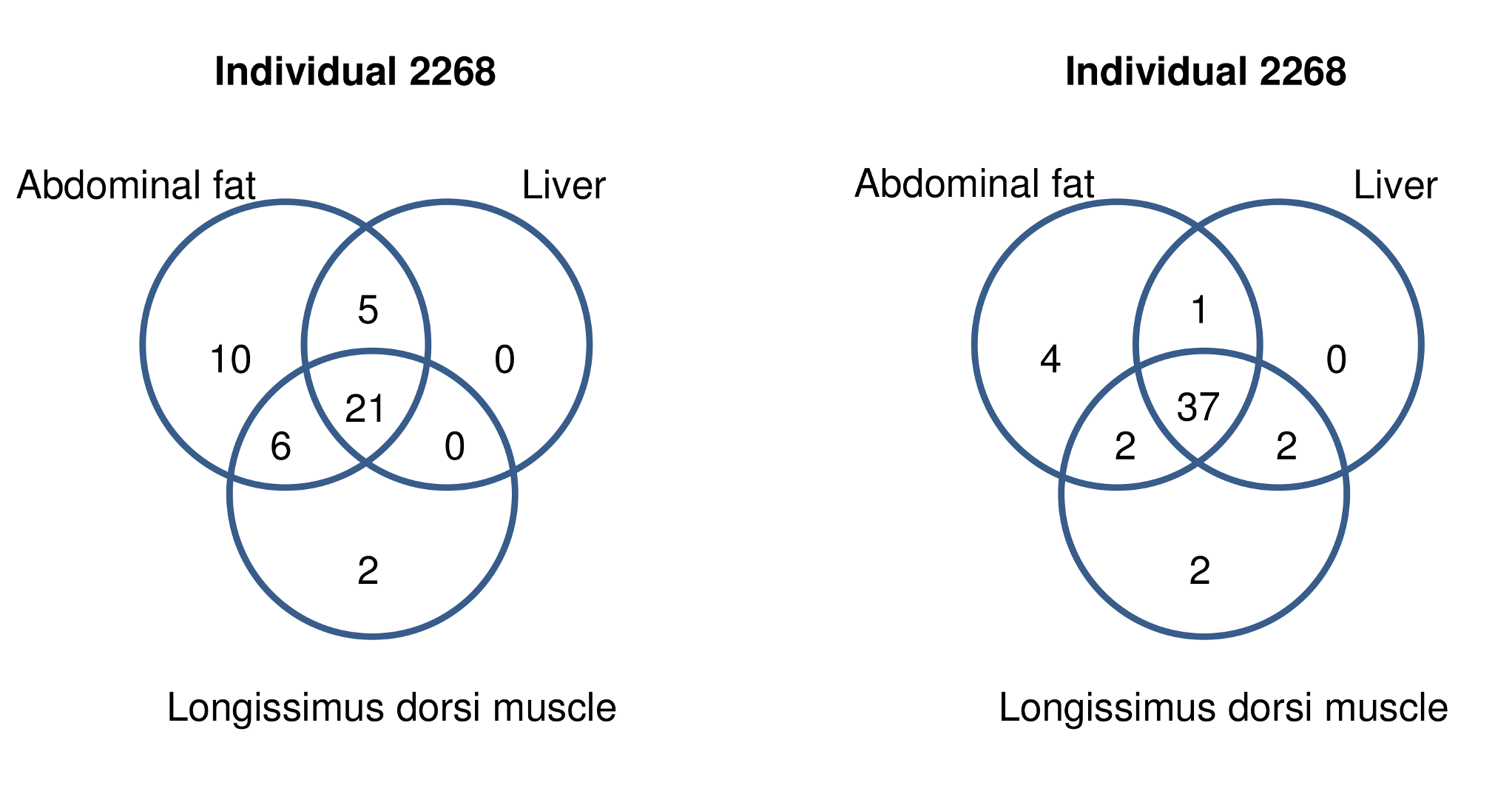

Supplement: Figure S1 — Venn diagrams of miRNA target sites predicted in pig 2268 and pig 2270. Predictions were performed on genes expressed in abdominal fat (AF), liver (LI) and longissimus dorsi muscle (LD) by TargetScan, PACMIT and TargetSpy algorithms. (TIFF) [file pone.0048607.s001.tiff]
